# Childhood vaccination in informal urban settlements in Nairobi, Kenya: Who gets vaccinated?

**DOI:** 10.1186/1471-2458-11-6

**Published:** 2011-01-04

**Authors:** Martin K Mutua, Elizabeth Kimani-Murage, Remare R Ettarh

**Affiliations:** 1 African Population and Health Research Center (APHRC), Shelter Afrique Center, Longonot Road, Upper Hill, Nairobi, Kenya

## Abstract

**Background:**

Recent trends in global vaccination coverage have shown increases with most countries reaching 90% DTP3 coverage in 2008, although pockets of undervaccination continue to persist in parts of sub-Saharan Africa particularly in the urban slums. The objectives of this study were to determine the vaccination status of children aged between 12-23 months living in two slums of Nairobi and to identify the risk factors associated with incomplete vaccination.

**Methods:**

The study was carried out as part of a longitudinal Maternal and Child Health study undertaken in Korogocho and Viwandani slums of Nairobi. These slums host the Nairobi Urban Health and Demographic Surveillance System (NUHDSS) run by the African Population and Health Research Centre (APHRC). All women from the NUHDSS area who gave birth since September 2006 were enrolled in the project and administered a questionnaire which asked about the vaccination history of their children. For the purpose of this study, we used data from 1848 children aged 12-23 months who were expected to have received all the WHO-recommended vaccinations. The vaccination details were collected during the first visit about four months after birth with follow-up visits repeated thereafter at four month intervals. Full vaccination was defined as receiving all the basic childhood vaccinations by the end of 24 months of life, whereas up-to-date (UTD) vaccination referred to receipt of BCG, OPV 1-3, DTP 1-3, and measles vaccinations within the first 12 months of life. All vaccination data were obtained from vaccination cards which were sighted during the household visit as well as by recall from mothers. Multivariate models were used to identify the risk factors associated with incomplete vaccination.

**Results:**

Measles coverage was substantially lower than that for the other vaccines when determined using only vaccination cards or in addition to maternal recall. Up-to-date (UTD) coverage with all vaccinations at 12 months was 41.3% and 51.8% with and without the birth dose of OPV, respectively. Full vaccination coverage (57.5%) was higher than up-to-date coverage (51.8%) at 12 months overall, and in both slum settlements, using data from cards. Multivariate analysis showed that household assets and expenditure, ethnicity, place of delivery, mother's level of education, age and parity were all predictors of full vaccination among children living in the slums.

**Conclusions:**

The findings show the extent to which children resident in slums are underserved with vaccination and indicate that service delivery of immunization services in the urban slums needs to be reassessed to ensure that all children are reached.

## Background

Immunization during childhood has been proven to be the most effective strategy for the prevention of many infectious diseases [1]. WHO estimates that as many as 2.5 million deaths among under-5 children worldwide are averted annually by immunization against diphtheria, tetanus, pertussis, and measles [2]. Recent estimates indicates that the global DTP3 immunization coverage of infants is 82%, and 23.5 million children did not receive DTP3 vaccine in 2008 [2]. Although the recent trend related to global vaccination coverage is positive with 120 countries reaching 90% DTP3 coverage in 2008, pockets of undervaccination continue to persist in parts of sub-Saharan Africa [3].

In Kenya, the proportion of children aged 12-23 months that are reported to have received all recommended vaccinations is 77.4% [4]. However, this proportion varies from 48.3% in the North Eastern Province to 85.8% in the Central Province. This geographical inequality in coverage reflects the variation in the influence of determinants of full vaccination across the different provinces. In Nairobi, 73% of children in this age range are reported to have received all vaccinations [4], but estimates in the slums within the city are usually much lower [5]. A study across the slums of Nairobi showed that full vaccination coverage of children was about 44% in these settlements compared to 73% for the whole of Nairobi [6]. Polio and measles vaccinations in these settlements were substantially lower than coverage in Nairobi, but slightly higher than that in the rural areas of Kenya, despite the overall immunization coverage being lower than in the rural areas [6]. Lower immunization coverage rates have also been observed in facilities that serve slums settlements in Nairobi and may be due to missed opportunities among clinic attendees and inappropriately administered vaccines [7].

Studies carried out in the Mathare slum of Nairobi have shown that parental age, marital status, level of education and poor knowledge about vaccinations are significantly associated with completion of the immunization schedule by under-5 children [8]. Other factors identified as predictors of incomplete vaccination among socio-economically disadvantaged children in the United States include lack of family support, lack of adequate prenatal care use, financial barriers and single motherhood [9,10]. The differences in living conditions between slum settlements suggest that the determinants of vaccination of children may be context specific and need to be studied in the different slums.

The objectives of this study were to determine extent of full and up-to-date (UTD) vaccination coverage among children aged between 12-23 months living in the slums of Korogocho and Viwandani, Nairobi, and to identify risk factors associated with incomplete vaccination in these resource-deprived urban settlements of Kenya.

## Methods

### Study area and population

The study was carried out in two informal settlements of Nairobi (Viwandani and Korogocho) where the African Population and Health Research Centre (APHRC) runs a demographic surveillance system referred to as the Nairobi Urban Health and Demographic Surveillance System (NUHDSS). The NUHDSS has been in operation since 2002 and has about 60,000 registered inhabitants in nearly 20,000 households. These two densely populated slums, each comprising 7 villages, have high unemployment, poverty, crime, poor sanitation and generally poorer health indicators when compared to Nairobi as a whole [5]. The two communities however have notable differences: Viwandani is bordered by an industrial area and attracts migrant workers with relatively higher education levels, while the population in Korogocho is more stable and shows more co-residence of spouses. In addition, Korogocho has less disparity with regard to sex and age distribution of the population compared with Viwandani. Being illegal settlements, the slums are served with limited health services. There are no public health facilities within the slums but there are public health facilities in the neighboring communities where residents of the slums can access vaccination services: Four health facilities are located in the neighbourhood of Korogocho and two are close to Viwandani. Vaccination services are also offered in private and non-governmental health facilities within or near the slums.

### Study Sample and Design

**Figure 1:**
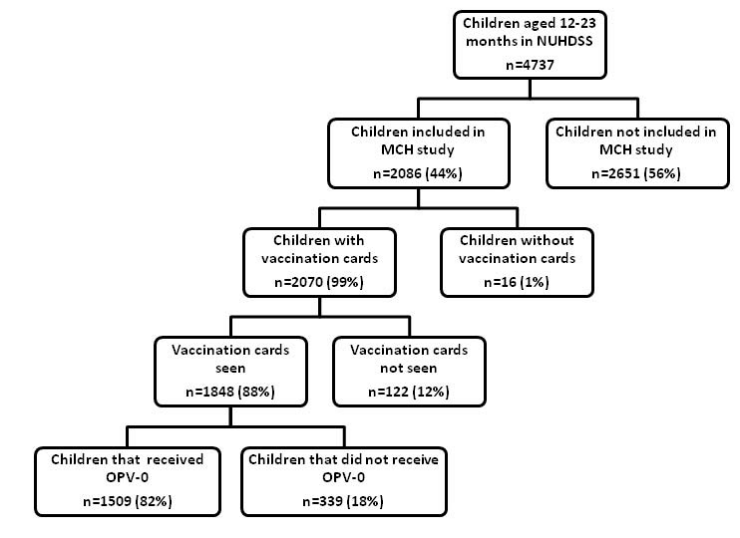
**Derivation of the sample of children included in the study from the NUHDSS**.

This study uses data from the Maternal and Child Health component of a broader project entitled "Urbanization, Poverty and Health Dynamics" being implemented in Korogocho and Viwandani. All women from the NUHDSS area who gave birth since September 2006 were enrolled in the project and administered a questionnaire which asked about the vaccination history of their children. For the purpose of this study, we used data on 1848 children aged 12-23 months who were expected to have received all the recommended vaccinations during the first 12 months after births. The vaccination details were collected during first visit about four months after birth with follow-up visits repeated thereafter at four month intervals. All vaccination data were obtained from vaccination cards which were sighted during the household visit, as well as by recall from mothers. Almost all of the children (99%) were said to have vaccination cards during the visits but only 88% (1848) of the cards were seen at the time of interview (Figure 1). Data on the socio-demographic characteristics of the households were also collected from the NUHDSS census rounds.

### Variables

We defined full vaccination status as receiving all the basic childhood vaccinations as recommended by WHO by the end of 24 months after birth. Vaccination status was considered UTD at 12 months if the child had received the following vaccinations in the first year of life: One dose of BCG received shortly after birth, three doses of triple vaccine for diphtheria, pertussis and tetanus (DTP) or pentavalent, three doses of polio (excluding OPV-0 given shortly after birth), received at 6, 10, and 14 weeks after birth respectively, and measles vaccinations at the age of 9 months. UTD vaccination coverage was determined for children after 3 months during which BCG, OPV 0,1 and 2, as well as DTP 1 and 2 would have been administered. Some children received polio vaccine at birth and, therefore the analyses were repeated with full vaccination including OPV-0. Analysis was conducted only for children aged 12-23 using data on their vaccination status obtained within the first 24 months after birth.

The household assets index was constructed using the principal component analysis (PCA). The assets index was derived from different assets owned by the household, both within the dwelling structure and elsewhere. These included motor vehicle, motorcycle, cooking stove, TV, refrigerator, and phone. The monthly expenditure was computed by dividing the monthly household expenditure by the equivalent household size, taking a child to be equivalent of half an adult. The poverty variables were computed at the household level and averaged for the village within which the households are located. The mean village poverty scores were then assigned to all the households in the respective villages and used as covariates in the modeling.

Nine covariates were included in the analysis: sex of child, maternal education (none, primary, and secondary or higher), maternal age at index child's birth (<20, 20-24, 25-29, 30+), parity (1, 2, and 3+), place of delivery (home or health facility), ethnicity (Kikuyu, Luhya, Luo, Kamba and Others), antenatal care (no ANC, seen a doctor, seen a nurse), birth weight (less than 2.5 kg, 2.5 kg or greater), postnatal care (no postnatal, postnatal care) and measures of poverty.

### Data Analysis

Descriptive analysis was used to show the characteristics of the participants in the study and the extent of coverage for specific, UTD and full vaccination. A bivariate model was fitted for all covariates and those with a *p *value of less than 0.25 were included in the multivariate analyses. Multivariate models were used to identify the risk factors associated with incomplete vaccination in the study settlements. The poverty variables were computed for each village and a multilevel (random intercept model) technique was used to account for these village level factors [11]. Due to multi-dimensionality of poverty measurements, we fitted separate models to assess the effects of poverty. All models were fitted with the STATA "xtlogit" or "xtmelogit" command using verified immunization status data from vaccination cards.

### Ethical considerations

The conduct of the Urbanization, Poverty and Health Dynamics programme of research was approved by the Ethical Review Board of the Kenya Medical Research Institute (KEMRI). The field workers were trained in research ethics and obtained informed consent from all respondents in the Maternal and Child Health project. The NUHDSS has also been approved by KEMRI's Ethical Review Board. Verbal consent is usually obtained from all respondents.

## Results

### Sample Characteristics

Of the total of 4737 children aged between 12-23 months resident in the slums during the period of the study, 1848 were included in the study by virtue of being born in the NUHDSS study area, were aged 12 months or above during the last visit, and also had their cards seen during the visits. Of the 1848 sampled, 875 were resident in Viwandani and 973 in Korogocho. The distribution by ethnicity varied between the two slums, and 85% of mothers in the study were married or in a civil union. About 71% of the children were delivered at health facility, and over 90% of mothers received antenatal and postnatal care. The other characteristics of the sampled children and their mothers are shown in Table 1.

**Table 1:** Socio-demographic characteristics of the study sample by slum of residence

	Korogocho	Viwandani	Total	
	
	%	%	N	%
Assets index*	-0.34 (0.24)	0.26 (0.11)	-	-
Expenditure (KSh)*	3386 (434)	3706 (279)	-	-
**Ethnicity of the Person**				
Kikuyu	34.5	21.9	480	28.1
Luhya	19.9	14.8	296	17.3
Luo	24.6	10.6	298	17.4
Kamba	6.0	35.3	359	21.0
Others	15.1	17.4	278	16.3
**Delivery place**				
Home	27.5	29.8	530	28.7
Health Facility	72.5	70.2	1316	71.3
**Marital status as at first interview**				
Not married	20.2	9.8	274	14.8
Married/In union	79.8	90.2	1572	85.2
**Mother's education**				
Incomplete Primary/No education	45.4	24.1	636	34.5
Completed Primary	39.6	43.7	770	41.7
Secondary	15.1	32.1	440	23.8
**Mother's age at delivery**				
<20	16.2	10.3	243	13.2
20-24	34.1	39.6	682	36.9
25-29	22.1	25.2	437	23.7
30+	27.7	24.9	484	26.2
**Parity**				
Parity 1	28.9	36.0	599	32.6
Parity 2	27.9	31.1	543	29.5
Parity 3+	43.2	33.0	698	37.9
**Antenatal care**				
No ANC	3.3	2.9	57	3.1
Seen a doctor	9.0	15.7	230	12.5
Seen a nurse	87.6	81.5	1559	84.5
**Postnatal care**				
No postnatal	8.5	1.9	94	5.1
Postnatal	91.5	98.1	1752	94.9
**Child's sex**				
Male	50.7	49.2	922	49.9
Female	49.8	50.8	924	50.1
**Birth weight**				
Less than 2.5 kg	12.8	13.3	234	12.7
2.5 kg or greater	87.9	86.7	1606	87.3

*These variables are calculated as mean (±SD) values for each slum settlement

### Vaccination Status

The coverage by vaccine and the proportion of children with up-to-date and full vaccination status are shown in Table 2. Vaccination coverage for BCG, which is administered immediately after birth, was 98.5% among all the children in the study. The coverage for the birth dose of OPV was lower than that for the other three OPV doses, and there was a slight decline in DTP/pentavalent coverage over the three doses from 97.5% to 96.9% with cards, or from 98.8% to 95.2% with recall included. Measles coverage was substantially lower than that for the other vaccines. Only 62.4% of children aged between 12-23 months had received verified measles vaccination with almost half of the children in Korogocho still unprotected against the disease. Up-to-date and full coverage were consistently higher in children when these were determined without inclusion of the birth dose of OPV. Up-to-date coverage rates at 12 months were 41.3% and 51.8% with and without the birth dose of OPV, respectively. Full vaccination coverage rates (57.5%) were higher than up-to-date coverage at 12 months overall, and in both slum settlements. In general, the coverage values for all vaccinations were slightly higher with the inclusion of maternal recall data.

**Table 2:** Specific, up-to-date and full vaccination coverage among children aged 12-23 months in Korogocho and Viwandani

Vaccination	Korogocho		Viwandani		Total			
	**Card + Recall**	**Card**	**Card + Recall**	**Card**	**Card + Recall**		**Card**	

	**%**	**%**	**%**	**%**	**N**	**%**	**N**	**%**

**BCG**	98.4	97.9	99.6	99.1	1830	99.0	1818	98.5
**Polio 0**	86.6	80.7	85.5	82.7	1590	86.0	1509	81.7
**Polio 1**	98.9	96.9	98.8	98.4	1827	98.9	1803	97.7
**Polio 2**	97.6	92.8	97.3	96.3	1800	97.4	1746	94.6
**Polio 3**	90.1	82.6	93.2	91.0	1694	91.7	1605	86.9
								
**DPT 1**	98.3	95.8	99.3	99.1	1826	98.8	1799	97.5
**DPT 2**	97.9	95.1	99.4	98.8	1823	98.7	1791	97.0
**DPT 3**	93.8	94.8	96.5	98.8	1759	95.2	1788	96.9
								
**Measles**	82.3	53.6	88.5	70.7	1580	85.5	1152	62.4
								
**UTD vaccination**								
**At 3 months**								
With OPV-0		43.9		62.7			989	53.6
Without OPV-0		56.9		75.2			1224	66.3
								
**At 12 months**								
With OPV-0		31.0		51.1			763	41.3
Without OPV-0		39.8		63.1			956	51.8
								
**Full vaccination**								
With OPV-0	63.8	39.6	68.5	55.0	1224	66.2	877	47.5
Without OPV-0	72.1	47.4	80.5	67.1	1412	76.4	1062	57.5

Values are mean (±SD)

### Timing of BCG and OPV-0 Vaccinations

The 13% disparity observed between the coverage for BCG and OPV-0 was investigated by examining the timing differences in the administration of both vaccines (Table 3). The average time to BCG and OPV-0 vaccinations were 12 and 13 days respectively. This duration was similar (11 days) even for children who received both vaccines on the same day. The mean durations to BCG and OPV-0 vaccination were greater when the two vaccines were administered on different dates, with BCG being administered 3 weeks after birth and OPV-0 being administered 4 weeks after birth.

**Table 3:** Timing of BCG and OPV-0 vaccinations

	BCG + OPV0 (n = 1448)	BCG + OPV-0 same date (n = 1251)	BCG + OPV-0 different dates (n = 197)	BCG only (n = 361)
Mean .time to BCG (days)	12.69 (25.96)	11.35 (24.58)	21.21 (32.26)	28.13 (40.61)
Mean time to OPV-0 (days)	13.83 (30.80)	11.35 (24.58)	29.52 (53.53)	-

### Bivariate Analysis

The sex and weight at birth of the child were not associated with full vaccination for children aged 12-23 months. Children born at a health facility were more likely to be fully vaccinated when this included OPV-0 compared with those born at home. Marital status of the mother was not significantly associated with full child vaccination, but mothers with primary level education or higher were more likely to have children who were fully full vaccinated. Maternal age and receipt of antenatal or postnatal care were associated with full vaccination of their children. Women with higher parity were less likely to have fully vaccinated children compared with those with lower parity. The slum of residence was also associated with vaccination status, with children in Viwandani being twice as likely to be fully vaccinated compared to those in Korogocho. The children of the Kikuyu ethnic group were more likely to be fully vaccinated compared to children of all other ethnic groups in the slums. The poverty variables used in this study were mean values calculated at village level. Household assets index and monthly expenditure were significantly associated with full vaccination of children when the birth dose of OPV was included (Table 4).

**Table 4:** Bivariate analysis of factors associated with full vaccination among children aged 12-23 months

	Including OPV-0		Excluding OPV-0		
	
	OR	95% CI	OR	95% CI	n
**Assets**	1.980**	[1.220,3.215]	1.924*	[1.098,3.372]	1735
**Expenditure**	1.000*	[1.000,1.001]	1.000*	[1.000,1.001]	1735
**Ethnicity **(Ref: Kikuyu)					
Luhya	0.459***	[0.336,0.627]	0.669**	[0.493,0.907]	1735
Luo	0.472***	[0.342,0.652]	0.793	[0.579,1.087]	
Kamba	0.795	[0.588,1.075]	1.058	[0.776,1.442]	
Others	0.608**	[0.445,0.832]	0.711*	[0.519,0.973]	
**Place of delivery **(Ref: Home)					
Health facility	1.542***	[1.233,1.927]	1.170	[0.939,1.458]	1735
**Marital Status **(Ref: Not in a Union)					
In a Union	0.763	[0.576,1.012]	0.963	[0.727,1.277]	1735
**Mother's education **(Ref: Incomplete Primary/No education)					
Completed Primary	1.475**	[1.158,1.880]	1.391**	[1.097,1.764]	1735
Secondary +	1.645***	[1.243,2.176]	1.517**	[1.148,2.006]	
**Mother's age **(Ref: <20)					
20-24	1.282#	[0.944,1.741]	1.233#	[0.912,1.666]	1735
25-29	1.291	[0.930,1.791]	1.136	[0.822,1.570]	
30+	1.135	[0.806,1.599]	1.026	[0.732,1.437]	
**Parity **(Ref: Parity 1)					
Parity 2	0.718**	[0.562,0.919]	0.686**	[0.534,0.881]	1735
Parity 3+	0.631***	[0.499,0.797]	0.614***	[0.484,0.778]	
**Antenatal care **(Ref: No ANC)					
Seen a Doctor	2.078*	[1.022,4.224]	1.658	[0.855,3.218]	1735
Seen a Nurse	2.101*	[1.084,4.074]	1.773	[0.963,3.263]	
**Postnatal care **(Ref: No postnatal care)					
Postnatal care	1.192#	[0.735,1.934]	1.446	[0.902,2.319]	1735
**Sex **(Ref: Male)					
Female	1.077	[0.887,1.308]	1.020	[0.839,1.240]	1735
**Slum **(Ref: Korogocho)					
Viwandani	2.395***	[1.971,2.910]	2.552***	[1.976,3.297]	1735
**Birth weight **(Ref: Less than 2.5 kg)					
2.5 kg or greater	0.904	[0.676,1.208]	0.840	[0.625,1.129]	1735

# p < 0.25; *p < 0.05; **p < 0.01; ***p < 0.001. All values are odds ratios [95% confidence intervals]

### Multivariate Analysis

The results of the multivariate analysis are shown in Table 5. The models included the variables that were significantly associated with full vaccination in the bivariate analysis. Some variables had missing information which led to the decrease in the number of observations used in the multivariate regression model as the method excludes the whole case if any information is missing. The regression model which included household assets showed that this dimension of poverty was associated with vaccination with or without the OPV-0. Poverty measured as household expenditure was also a predictor of full childhood vaccination. Maternal level of education was a significant determinant of full vaccination when OPV-0 was included, with children of mothers who had completed primary education having close to one and a half times higher odds of being vaccinated compared to those of mothers with no education (p < 0.05). This association was present in both models. Similarly, maternal age was a strong predictor of vaccination of a child with older mothers being more likely to have children who were vaccinated compared with mothers who were aged less than 20 years. Whereas children born at the health facility had 1.3 times higher odds of full vaccination when the birth dose was included, maternal attendance for antenatal and postnatal care was not significantly associated with full vaccination in children. As seen in the bivariate analysis, higher maternal parity was associated with a lower likelihood of full vaccination among children, when both including and excluding the polio birth dose. Ethnicity was also significantly associated with full vaccination, with the Luhya, Luo and other ethnic groups having lower odds of vaccination compared to kikuyu children, but mostly when the outcome included the polio birth dose.

**Table 5:** Multivariate analysis of determinants of full vaccination among children aged 12-23 months

	Assets				Expenditure			
	
	With OPV-0		Without OPV-0		With OPV-0		Without OPV-0	
	
	OR	95% CI	OR	95% CI	OR	95% CI	OR	95% CI
								
**Poverty**	1.992**	[1.314,3.018]	1.821*	[1.107,2.996]	1.005**	[1.002,1.008]	1.004*	[1.000,1.007]
**Ethnicity **(Ref: Kikuyu)								
Luhya	0.529***	[0.384,0.728]	0.743	[0.542,1.018]	0.538***	[0.390,0.742]	0.753	[0.549,1.030]
Luo	0.546***	[0.392,0.760]	0.885	[0.638,1.227]	0.549***	[0.392,0.767]	0.888	[0.639,1.234]
Kamba	0.833	[0.614,1.131]	1.074	[0.784,1.471]	0.845	[0.621,1.150]	1.085	[0.791,1.489]
Others	0.664*	[0.482,0.916]	0.795	[0.575,1.098]	0.688*	[0.498,0.951]	0.809	[0.584,1.119]
**Place of delivery **(Ref: Home)								
Health facility	1.258	[0.990,1.597]	1.028	[0.812,1.301]	1.274*	[1.002,1.619]	1.035	[0.817,1.311]
**Mother's education **(Ref: Incomplete primary/No education)								
Completed primary	1.3024*	[1.011,1.676]	1.224	[0.957,1.565]	1.311*	[1.017,1.689]	1.23	[0.961,1.573]
Secondary +	1.3089	[0.976,1.754]	1.262	[0.943,1.687]	1.334	[0.994,1.790]	1.277	[0.954,1.709]
**Mother's age **(Ref: <20)								
20-24	1.4831*	[1.057,2.079]	1.539*	[1.102,2.150]	1.512*	[1.077,2.123]	1.556**	[1.114,2.175]
25-29	1.7599**	[1.181,2.622]	1.705**	[1.148,2.533]	1.798**	[1.205,2.683]	1.722**	[1.158,2.561]
30+	1.7307*	[1.115,2.686]	1.748*	[1.132,2.700]	1.760*	[1.132,2.738]	1.764*	[1.141,2.727]
**Parity **(Ref: Parity 1)								
Parity 2	0.658**	[0.500,0.867]	0.606***	[0.458,0.802]	0.653**	[0.496,0.861]	0.604***	[0.456,0.795]
Parity 3	0.5634***	[0.408,0.776]	0.520***	[0.376,0.719]	0.561***	[0.406,0.774]	0.518***	[0.374,0.718]
**Antenatal care **(Ref: No ANC)								
Seen a Doctor	1.5036	[0.721,3.132]	1.383	[0.697,2.744]	1.597	[0.765,3.333]	1.448	[0.729,2.878]
Seen a Nurse	1.5503	[0.782,3.070]	1.482	[0.791,2.779]	1.578	[0.796,3.126]	1.494	[0.797,2.802]
**Postnatal care **(Ref: No postnatal								
Postnatal care	1.1391	[0.693,1.872]	1.376	[0.849,2.229]	1.122	[0.680,1.851]	1.362	[0.839,2.210]
**Sex **(Ref: Male)								
Female	1.0698	[0.876,1.305]	1.005	[0.824,1.229]	1.068	[0.875,1.304]	1.004	[0.823,1.224]
								
**AIC**	2280.46		2300.48		2279.53		2299.88	
**n**	1735		1735		1735		1735	

*p < 0.05; **p < 0.01; ***p < 0.001. All values are adjusted odds ratios [95% confidence intervals].

## Discussion

This study describes immunization coverage and risk factors for incomplete vaccination for children aged 12-23 months in urban informal settlements in Nairobi. The study shows poor full immunization coverage according to WHO recommendations for full vaccination at 58%. Immunization coverage was particularly low for measles at 62%. The study also indicates even poorer up-to-date immunization coverage at only 52%. Significant determinants of complete vaccination in these communities included poverty, ethnicity, place of delivery, mother's education and parity of the mother. These results clearly indicate important areas for intervention to improve immunization coverage among the urban poor in sub-Saharan Africa.

The full immunization coverage for children aged 12-23 months reported from vaccination cards in this study is low compared to that reported nationally, in urban areas and even in rural areas in Kenya. According to the 2008-2009 Kenya Demographic and Health Survey, the national coverage for full immunization is 77%, while that of Nairobi as a whole is 73%. Urban areas in Kenya have the highest level at 81% while that of rural areas is 76% [4]. The results in this study indicate that children living in urban informal settlements are the most disadvantaged sub-group and do not benefit from the urban advantage. While there is an improvement from results of a study carried out in the Nairobi slums in 2000 which showed that full immunization coverage was 44%, the improvement is small compared to that from 57% to 77% reported nationally for Kenya between 2003 and 2008 [4,12]. The full immunization coverage compares with that of other slum areas in developing countries [13,14]. For example, while full immunization coverage among children aged 12-23 months in Bangladesh as a whole is 75%, only 54% of children of the same age are fully immunized in the urban slums of Dhaka [14].

Various reasons may explain the lower levels of full immunization coverage in urban slums in the developing world. A comprehensive review of immunization services in urban areas in developing countries identified several challenges that are unique to urban areas [15]. These include rapid population growth particularly in slum populations, array of types of service providers in both private and public sectors, other more pressing challenges that need to be prioritized and the need to use creative strategies to reach marginal sub-populations [15]. Similar challenges face the urban poor populations in Nairobi. In addition, being illegal settlements, slum areas in Nairobi have in the past had little recognition from the government and therefore have been marginalized with regards to provision of basic services. Although immunization services are offered free of charge at public health facilities, these facilities are often not found in the slums and slum dwellers have to seek for public services outside of the slum areas. They otherwise seek services offered by private practitioners in the slums who are mostly illegal and offer sub-standard and relatively expensive services [16]. This limits access to basic services such as vaccination services and may be the main reason for low full immunization coverage.

While achieving high immunization coverage against vaccine-preventable diseases is of significant public health importance, achieving a high level of UTD vaccination coverage may even have higher-reaching benefits. Using vaccination card data, this study indicates a lower level of UTD vaccination than that of full coverage, declining from 66% for vaccinations that should be completed by 3 months including BCG, polio 1, 2, and DTP/pentavalent 1, 2, to 52% at 12 months when all vaccines should have been given. Evidence indicates that giving vaccines at the wrong time may have adverse implications [17]. For example, studies in West African countries indicate that DTP given concurrently with or after measles vaccine is associated with higher mortality than having measles vaccine alone as the latest vaccine [18]. Further, a study in Bangladesh showed that giving BCG and DTP after the age of measles vaccine increased mortality by three-fold compared to not vaccinating at all [19]. This indicates the importance of emphasizing immunization on schedule for children living in informal settlements.

The study indicates high vaccination coverage for vaccines that are given within the first few months after birth at between 87% and 98%. On the other hand, coverage for measles, given towards the end of the first year was poor with only 62% receiving the vaccine. This difference in coverage between these two sets of vaccines has also been documented in other studies in developing countries, [13,20,21] and may be due to the long interval between them. The low immunization coverage for measles is of particular public health concern given both the specific and non-specific beneficial effects of measles vaccine on childhood morbidity and mortality documented in several studies in the developing world [22,23]. The other concern with the low coverage of measles vaccination is the absence of herd immunity. High measles coverage provides herd immunity thereby decreasing the risk for measles exposure and affording protection to the small proportion of individuals who are not vaccinated.

This study also reveals the extent of missed opportunities for vaccination in the slum settlements. For example, while BCG vaccination coverage is almost 100%, close to 20% of children do not receive the zero dose of polio given at birth together with BCG. Although the birth dose of polio is not within the WHO guidelines for full immunization, it is important for the efforts towards polio eradication. In addition, while only 3% are not given DTP3/pentavalent3, 13% are not given OPV 3 dose given at the same time. A few explanations may be given for the disparities: evidence in other slums indicates lack of confidence of health workers in administering two vaccines at the same time owing to fear of contraindication. Furthermore, shortage of vaccines and the unwillingness of health workers to open vaccine vials if there are not enough children needing the vaccine at a given time have been cited as reasons for these differences [24,25]. The results indicate significant delays in BCG and OPV-0 vaccinations immediately after birth. The delay of 11 days observed suggests that supply of the vaccines at health facilities which conduct deliveries may not always be assured at the time of birth. The delay may also be associated with mothers who deliver outside of formal health facilities but are aware of the need for these vaccines and take the children for vaccination at health facilities a few days later. The substantial delay observed when the two vaccines are administered at different dates or when only BCG was given suggests that vaccine shortage at the health facilities at time of delivery problems is a major factor in the delay or non-receipt of BCG or OPV-0 vaccinations. These challenges with vaccine stockouts are common among the numerous privately run health facilities in Korogocho and Viwandani.

From the experience working in the community, mothers often report shortage of the polio vaccine. This irregularity is not often reported with regards to BCG and DTP/Pentavalent. With regards to OPV zero, there is a duration limit of 14 days within which the vaccine should be given, if children report after these 14 days, they are given OPV 1 instead. The higher coverage for DTP3/pentavalent compared to OPV3 may be due to donor emphasis on using DTP3/pentavalent to monitor and evaluate performance of vaccination programs. However, there is need for further investigations as there seems to be no disparity between OPV 1 and 2 and the respective DTP/pentavalent doses. These findings indicate a need for increased awareness amongst health care workers to ensure administration of all eligible vaccines at the time the caregiver visits the clinic for vaccination of a child. Further, the government may need to ensure regular supply of polio vaccine.

The finding of greater likelihood of full vaccination among children born at a health facility has been reported in previous studies [26,27]. Mothers who deliver at health facilities may be more frequent users of health facilities and services including immunization for children. The administration of BCG and OPV at birth is required at registered health facilities and may partly account for the higher proportion of facility-delivered children with full vaccination. Home deliveries in urban slums and rural areas are often conducted by traditional birth attendants and usually do not involve administration of vaccines [27]. Other studies have shown that maternal education, attendance for antenatal and postnatal care, and parity are associated with full vaccination among children [8,28,29]. In this study, women who received antenatal and postnatal care were not significantly more likely to have a fully vaccinated child compared to those who did not. A high proportion of women in the two slums reported utilizing antenatal and postnatal services. However, the quality of antenatal and postnatal care services provided at health facilities serving the slum population is generally substandard and may not include components such as immunization which these services are expected to provide [30]. The study also showed that mothers resident in the slums who had higher parity were less likely to ensure that their children received all the required vaccinations. This relationship has been shown in other studies in non-slum areas of Kenya and has been linked to the higher cost and demands on resources caused by having more children, which may adversely affect healthcare utilization [29,31]. The greater vaccination coverage among children resident in Viwandani suggests the presence of contextual factors within the slum that influence the decision of caregivers to get their children vaccinated. Such contextual factors may include level of education in the slums (maternal, paternal and other household members). The higher vaccination coverage in Viwandani may be because this area attracts migrant workers with relatively higher education levels, which may influence vaccination coverage at the community level. Additionally, given that public health facilities where slum residents may seek vaccination services are located outside the slums, Korogocho residents, being of lower socio-economic status may be limited by transport costs. Within both slums, greater likelihood of full childhood vaccination was associated with being of the Kikuyu ethnic group. This is consistent with previous reports for the whole of Kenya in which the Kikuyu children or children from Central Province where this ethnic group mainly lives had the highest odds of being fully vaccinated or being at advantage with regards to other health outcomes [4,32]. This suggests positive cultural characteristics of this group in relation to vaccination and health seeking behavior in general. It may also relate to higher socio-economic status or education attainment among Kikuyus (or people from Central Province) documented nationally [4]. Anecdotal evidence from the NUHDSS data also suggests that Kikuyus have higher social economic status in the slums. However, further research may need to unravel the ethnic disparities with regards to vaccination.

The poverty variables used in the study included two measures of poverty, namely household assets and monthly expenditure. These variables reflect different dimensions of poverty in the slums. Household assets is a slow-changing measure of poverty and was associated with full vaccination. Children in villages with greater household assets and expenditure were more likely to be fully vaccinated than those in villages with less household assets. Assets are a reliable measure of the underlying structural well-being of a household whereas household expenditure demonstrates much greater periodic fluctuation [33]. This distinction may explain the extent of association of these measures with full vaccination of children in the slum. There were similarities in the odds ratios of the variables in the two models using each measure of poverty, suggesting that household assets and monthly expenditure are correlated in the slums and do not individually alter the association of the various factors with the likelihood of vaccination. Financial barriers among the socio-economically disadvantaged groups have been shown to predict under-vaccination although these are typically represented as costs or relative measures such as wealth quintiles [34-36]. This study, which explores specific dimensions of poverty in the resource-deprived slums of Nairobi, demonstrates that household economic deprivation when expressed at community level influences the likelihood of full childhood vaccination.

This study has a number of strengths and limitations. Vaccination status was determined using data from vaccination cards thereby ensuring the accuracy of information. This eliminates the recall bias that exists in cases where vaccination cards are unavailable and researchers have to rely on the mother's report. Only a small proportion of children (16%) did not have vaccination cards, compared to 30% nationally and 58% for Nairobi Province reported in the 2008-2009 Kenya Demographic and Health Survey [4]; these were omitted from the regression analysis. The longitudinal nature of the study ensured timely updating of the vaccination data, hence reducing the bias that would have otherwise been created by loss of vaccination records. The inclusion of children born throughout the duration of the MCH study ensured a larger sample size but assumes that the influence of maternal factors on vaccination status is the same throughout the study period.

## Conclusions

The importance of immunization against the killer diseases in the developing world cannot be overemphasized. The low full immunization coverage documented in this study among children in the urban informal settlements of Nairobi, particularly for measles vaccination, indicates the need for strategies to address the situation. The results also indicate the need to emphasize timely administration of vaccines, given the documented consequences of administering vaccines at the wrong time. Vigilance amongst health professionals should also be enhanced through awareness creation to minimize missed opportunities for administration of the different types of vaccination expected to be given concurrently when the mother presents at the clinic. This study has also identified specific areas for intervention as far as factors associated with lower vaccination are concerned. Programmes targeting mothers of lower socio-economic status such as those with no education, those in most poor households and with many children are required. Such programmes may include health education and immunization campaigns at the community level to improve coverage. Awareness concerning delivery at health facilities should also be created among the slum population as this is associated with higher likelihood of childhood immunization, especially for vaccinations administered at birth.

## Competing interests

The authors declare that they have no competing interests.

## Authors' contributions

MKM: participated in the study design, supervised study implementation, performed analysis and interpretation of the data, contributed in writing the manuscript. EK-M, RRE: participated in the study design, performed interpretation of the data, and contributed in writing and revising the paper critically. All authors read and approved the final manuscript.

## Pre-publication history

The pre-publication history for this paper can be accessed here:

http://www.biomedcentral.com/1471-2458/11/6/prepub
